# Supplementary material for: The Pulvinus Is the Weak Point for Stem Lodging Resistance in Ripe Barley
Source: Plants (Basel). 2024 Nov 12;13(22):3172. doi: 10.3390/plants13223172 (PMC11597801; doi:10.3390/plants13223172)
Supplement: Supplementary file 1 [file plants-13-03172-s001.zip › plants-3280301-supplementary.pdf]

Stem state=dry

7 Variables:	re re-ri I E EI sb BS

Pearson Correlation Coefficients, N = 120
Prob > |r| under H0: Rho=0

	re	re-ri	I	E	EI	sb	BS
re<br>re	1.00000	0.70572<br><.0001	0.96442<br><.0001	-0.60083<br><.0001	0.64089<br><.0001	0.12007<br>0.1915	0.86058<br><.0001
re-ri<br>re-ri	0.70572<br><.0001	1.00000	0.75406<br><.0001	-0.35595<br><.0001	0.65679<br><.0001	0.19722<br>0.0308	0.76240<br><.0001
I<br>I	0.96442<br><.0001	0.75406<br><.0001	1.00000	-0.58498<br><.0001	0.61019<br><.0001	0.06215<br>0.5001	0.84135<br><.0001
E<br>E	-0.60083<br><.0001	-0.35595<br><.0001	-0.58498<br><.0001	1.00000	0.16076<br>0.0794	0.09426<br>0.3058	-0.43661<br><.0001
EI<br>EI	0.64089<br><.0001	0.65679<br><.0001	0.61019<br><.0001	0.16076<br>0.0794	1.00000	0.28392<br>0.0017	0.67432<br><.0001
sb<br>sb	0.12007<br>0.1915	0.19722<br>0.0308	0.06215<br>0.5001	0.09426<br>0.3058	0.28392<br>0.0017	1.00000	0.55474<br><.0001
BS<br>BS	0.86058<br><.0001	0.76240<br><.0001	0.84135<br><.0001	-0.43661<br><.0001	0.67432<br><.0001	0.55474<br><.0001	1.00000

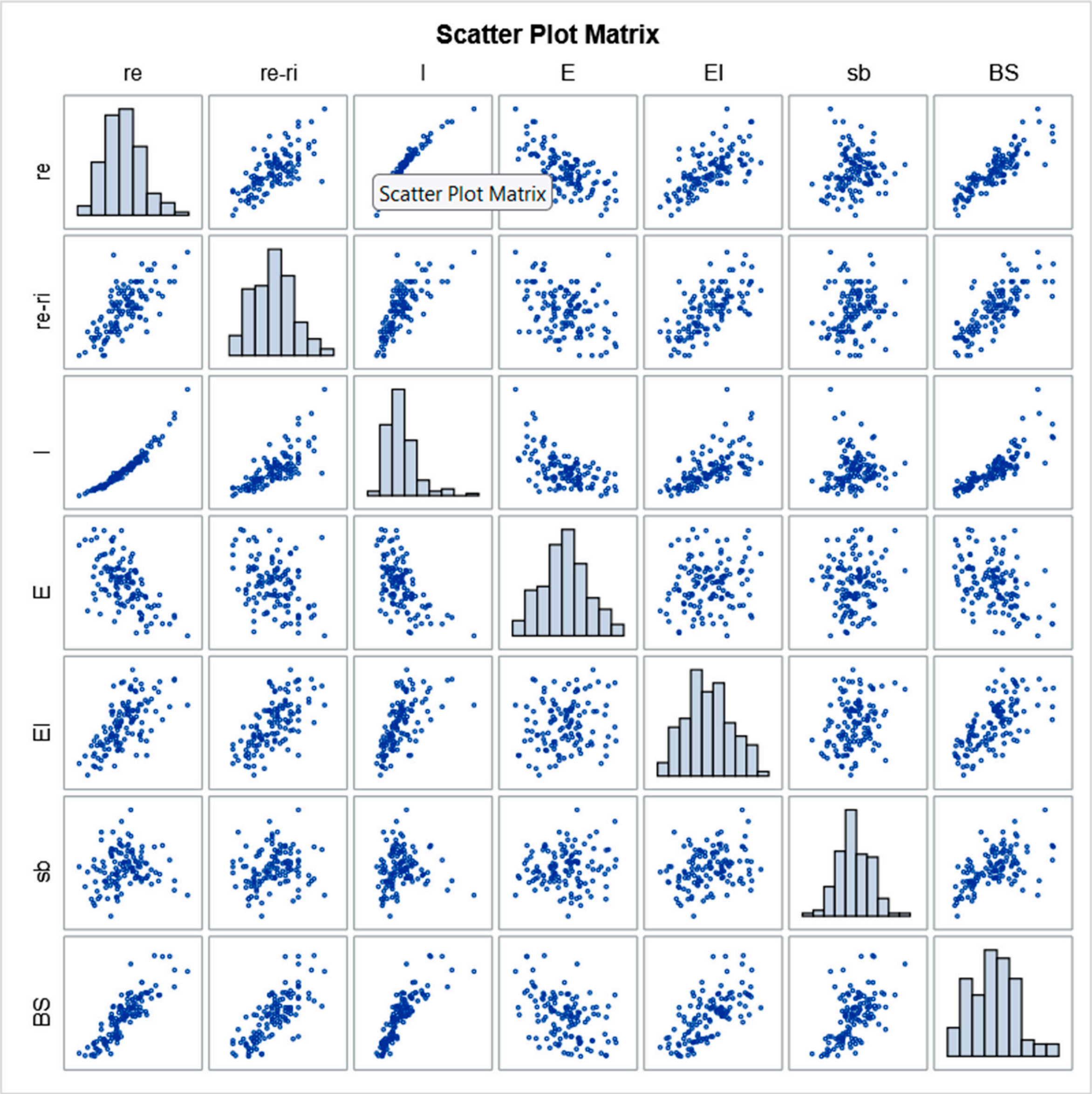
Scatter Plot Matrix
re
re-ri
I
E
EI
sb
BS
Scatter Plot Matrix

Stem state=wet

7 Variables:	re re-ri I E EI sb BS

Pearson Correlation Coefficients, N = 110<br>Prob > |r| under H0: Rho=0							
	re	re-ri	I	E	EI	sb	BS
re<br>re	1.00000	0.76771<br><.0001	0.97058<br><.0001	-0.60119<br><.0001	0.69758<br><.0001	0.32029<br>0.0006	0.82645<br><.0001
re-ri<br>re-ri	0.76771<br><.0001	1.00000	0.83197<br><.0001	-0.46120<br><.0001	0.63051<br><.0001	0.29883<br>0.0015	0.74527<br><.0001
I<br>I	0.97058<br><.0001	0.83197<br><.0001	1.00000	-0.57889<br><.0001	0.69547<br><.0001	0.30756<br>0.0011	0.84682<br><.0001
E<br>E	-0.60119<br><.0001	-0.46120<br><.0001	-0.57889<br><.0001	1.00000	0.09725<br>0.3122	0.10927<br>0.2558	-0.33742<br>0.0003
EI<br>EI	0.69758<br><.0001	0.63051<br><.0001	0.69547<br><.0001	0.09725<br>0.3122	1.00000	0.50280<br><.0001	0.76475<br><.0001
sb<br>sb	0.32029<br>0.0006	0.29883<br>0.0015	0.30756<br>0.0011	0.10927<br>0.2558	0.50280<br><.0001	1.00000	0.74171<br><.0001
BS<br>BS	0.82645<br><.0001	0.74527<br><.0001	0.84682<br><.0001	-0.33742<br>0.0003	0.76475<br><.0001	0.74171<br><.0001	1.00000

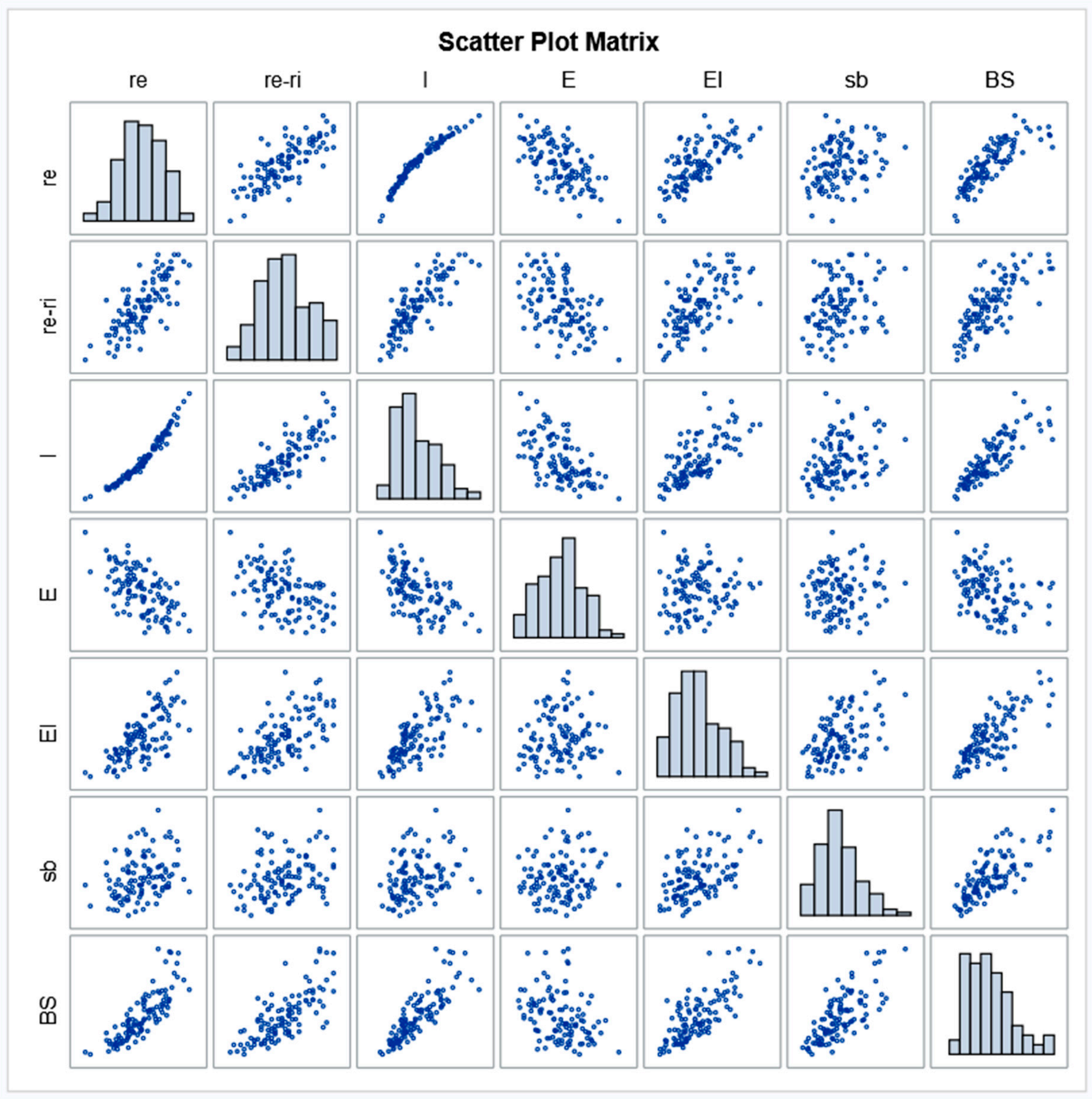
Scatter Plot Matrix
re
re-ri
I
E
EI
sb
BS
